# Pretreatment immune-inflammatory prognostic score in predicting clinical outcomes in esophageal squamous cell carcinoma receiving neoadjuvant immunochemotherapy

**DOI:** 10.3389/fimmu.2025.1617681

**Published:** 2025-08-06

**Authors:** Qiang Zhao, Liang Wang, Xun Yang, Jifeng Feng, Qixun Chen

**Affiliations:** ^1^ Department of Thoracic Surgery, Zhejiang Cancer Hospital, Hangzhou Institute of Medicine (HIM), Chinese Academy of Sciences, Zhejiang, Hangzhou, China; ^2^ Key Laboratory Diagnosis and Treatment Technology on Thoracic Oncology, Zhejiang Cancer Hospital, Zhejiang, Hangzhou, China

**Keywords:** esophageal squamous cell carcinoma, immune-inflammatory prognostic score, neoadjuvant immunochemotherapy, prognosis, survival

## Abstract

**Purpose:**

In this research, we established, for the first time, an immune-inflammatory prognostic score (IIPS) reflecting the balance of immune and inflammatory status to explore its prognostic value in patients with esophageal squamous cell carcinoma (ESCC) receiving neoadjuvant immunochemotherapy (NICT).

**Methods:**

In this retrospective study, two hundred and five ESCC patients who received NICT were included. To ascertain whether IIPS was superior to other traditional immune-inflammatory indices (IIIs), we compared their predictive values. The association between IIPS and overall survival (OS)/disease-free survival (DFS) was also investigated. For survival analyses, the Kaplan-Meier method and Cox proportional hazard regression analyses were employed.

**Results:**

With a mean age of 64 years (range: 45–75 years), there were 181 (88.3%) males and 24 (11.7%) females. Sixty-four (31.2%) patients achieved pCR after NICT. A total of 79 (38.5%) patients relapsed, and 55 (26.8%) cases died. The connection between DFS/OS and IIPS suggested that their interaction was non-linear. The restricted cubic spline (RCS) model identified 200 as the ideal cutoff point for IIPS. Patients exhibiting high IIPS demonstrated significantly worse 3-year OS (63.7% vs. 82.5%, P =0.002) and DFS (47.1% vs. 75.7%, P <0.001) compared to those with low IIPS. Based on the results of the Cox regression analyses, IIPS was a predictor of OS (hazard ratio [HR] =1.864, 95% CI =1.053-3.301, P =0.033) and DFS (HR =2.225, 95% CI =1.376-3.597, P =0.001).

**Conclusion:**

The treatment efficacy of NICT for ESCC can be predicted by pretreatment IIPS. IIPS is an innovative, sensitive, and useful index that helps clinicians giving individualized treatments because of improved prognostic stratification.

## Introduction

Esophageal cancer persists as a formidable global health challenge, ranking among the most prevalent digestive malignancies worldwide (1). Significant geographical differences in disease burden are revealed by recent epidemiological investigations, with high-incidence locations showing incidence rates 20-fold higher than low-risk areas (2). This malignancy demonstrates distinct histopathological stratification, comprising two predominant subtypes: adenocarcinoma and esophageal squamous cell carcinoma (ESCC). The prognosis is still poor even with significant improvements in multidisciplinary therapies such as neoadjuvant chemoradiotherapy (NCRT) or neoadjuvant chemotherapy (NCT) (3). It is worth noting that the prognosis of patients with advanced EC has been greatly impacted by immunotherapy, which has appeared as a viable treatment option in recent years (4, 5). Furthermore, neoadjuvant immunochemotherapy (NICT) appears to be safe and efficacious for locally advanced EC, according to accumulating evidence (6–8). The clinical effectiveness of NICT in EC must be confirmed, nevertheless, by additional validation.

The balance between the host inflammatory response and immune status is reflected by immune-inflammatory indices (IIIs), such as C-reactive protein (CRP), albumin (ALB), lymphocytes (LYMs), neutrophils (NEUs), monocytes (MONs), and platelets (PLTs), which has drawn a lot of interest in recent years. It is becoming more widely acknowledged that these indicators are essential to the prognosis of cancer (9, 10). Furthermore, other commonly used indicators, such as the NEU to LYM ratio (NLR), PLT to LYM ratio (PLR), LYM to MON ratio (LMR), CRP to ALB ratio (CAR), systemic inflammation response index (SIRI), and systemic immune-inflammation index (SII), have also been demonstrated to have prognostic value in various cancers, including EC (11–15). The majority of these indicators listed above, however, only have two or three of these IIIs. Additionally, the utility of these hematological indices in clinical practice is restricted due to their poor discriminative capabilities and contentious outcomes.

To date, sensitive and useful hematological indices to forecast the treatment results in EC receiving NICT are currently lacking. Additionally, the clinical results have received increased attention due to the extensive use of NICT in the treatment of EC. Since cancer, immunity, and inflammation are thought to interact in complex ways, using more composite indices that represent the global immune-inflammatory status may lead to more accurate clinical results. In this research, we established, for the first time, an immune-inflammatory prognostic score (IIPS) reflecting the balance of immune and inflammatory status to explore its prognostic value in patients with ESCC receiving NICT.

## Materials and methods

### Patient selection

**Figure 1:**
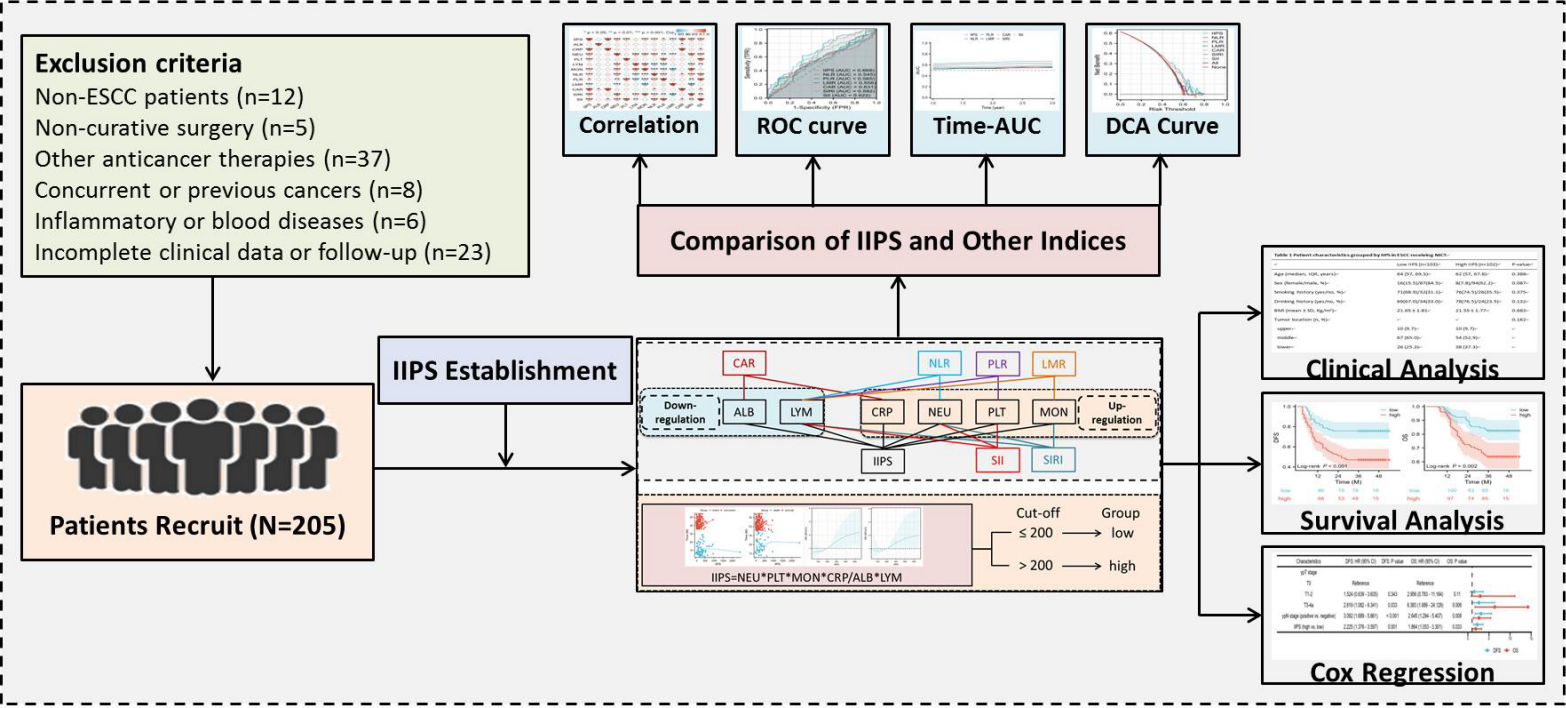
The recruitment process used in the current study.

This retrospective analysis enrolled ESCC patients who underwent NICT between 2019 and 2021. Hematological indicators and clinical information were gathered from the patients. The study protocol received approval from the Ethics Committee of Zhejiang Cancer Hospital (IRB2020320) and adhered to the principles outlined in the Declaration of Helsinki. Exclusion criteria were applied as specified: (1) patients who had other pathological forms of EC; (2) patients who were also receiving other anticancer treatments; (3) patients who had non-radical resection after NICT; (4) patients who had missing follow-up time or incomplete data; (5) patients with other autoimmune, hematologic, or inflammatory diseases; or (6) patients who had other malignancies at the time or in the past. The recruitment procedure followed in the current investigation is depicted in **Figure 1**. In this study, tumor staging followed the 8th edition AJCC/UICC TNM classification system (16).

### Treatment and follow-up

Prior to surgery, each patient underwent two NICT cycles every 21 days. On day 1, either camrelizumab (200 mg), sintilimab (200 mg), tislelizumab (200 mg), pembrolizumab (2 mg/Kg), or nivolumab (2 mg/Kg) was given. On days 1 and 8, albumin-bound paclitaxel (120 mg/m^2^) was given; on day 1, carboplatin (area under the curve = 5 mg/ml/min) was given. Surgery using the Ivor Lewis or McKeown method was often planned to take place four to six weeks following the conclusion of the previous NICT (17). There is currently no agreement regarding adjuvant treatment. Adjuvant immunotherapy following NCRT may be beneficial for patients, according to the CheckMate 577 research (18). Therefore, after radical resection, adjuvant therapy was given; however, it was not mandatory, especially for patients whose postoperative pathology results included ypN1–3 and/or ypT3/T4a. The last time to follow up will be in December 2024.

### IIPS and other hematological IIIs

**Figure 2:**
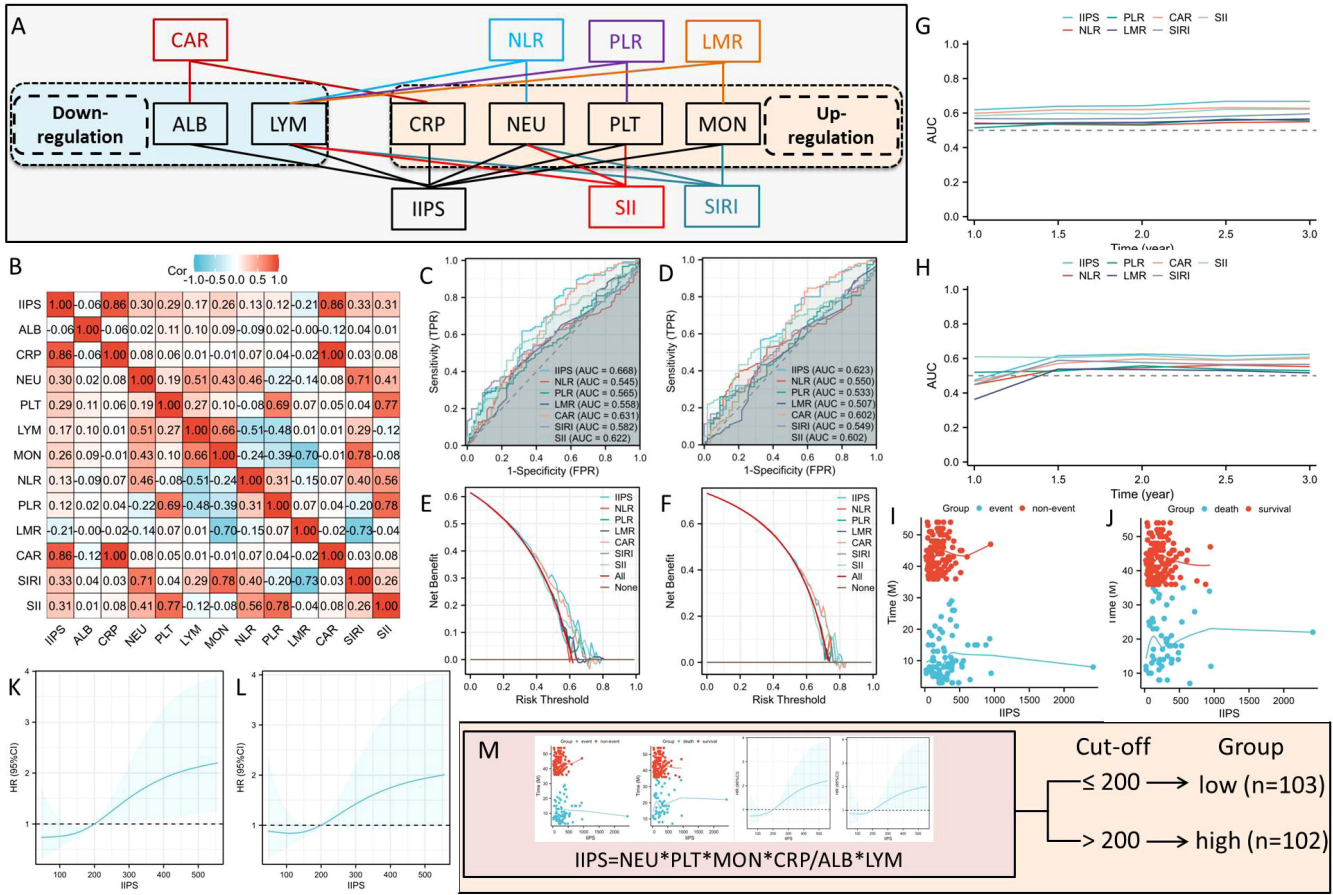
The links between the various IIIs and their composition **(A)**. The correlation diagram **(B)** of hematological IIIs. ROC analyses in DFS **(C)** and OS **(D)**. DCA analyses in DFS **(E)** and OS **(F)**. Analyses of time-dependent AUCs in DFS **(G)** and OS **(H)**. IIPS has a non-linear connection with OS **(J)** and DFS **(I)**. The optimal IIPS threshold in DFS **(K)** and OS **(L)** by RCS. Patients were split into two groups **(M)**.

Clinical data, including baseline characteristics, post-treatment pathological staging (ypT and ypN stages), and a variety of pretreatment hematological indices, including CRP, ALB, NEUs, MONs, LYMs, and PLTs, were extracted from electronic medical records. **Figure 2A** illustrates the links between the various IIIs and their composition. As is known from **Figure 2A**, among the above six hematological indicators, two IIIs (ALB and LYM) are down-regulated, while the other four IIIs (CRP, NEU, MON, and PLT) are up-regulated. The IIPS were calculated using the following formulas: IIPS= CRP × PLT × NEU × MON/LYM × ALB (**Figure 2A**).

### Statistical analysis

Statistical analyses included one-way ANOVA for continuous variables and Fisher’s exact or chi-square tests for categorical variables. The restricted cubic spline (RCS) was used to identify the ideal thresholds for IIPS based on the examination of the non-linear connection between IIPS and disease-free survival (DFS)/overall survival (OS). Decision curve analyses (DCAs), time-dependent areas under the curves (AUCs), and receiver operator characteristic curves (ROCs) were used to compare and assess the clinical applicability and AUCs of IIPS and other IIIs. The predictors of DFS and OS were assessed using the Cox proportional risk model, which included both univariate and multivariate analyses. The statistically significant components identified in univariate analysis were then shared in multivariate analysis using stepwise regression. Cox proportional hazards models, expressed as hazard ratios (HRs) with 95% confidence intervals (CIs), were employed to evaluate DFS and OS. A significance threshold of *P* < 0.05 was applied, with analyses performed using R 4.1.2 and SPSS 20.0.

## Results

### Patient characteristics

Two hundred and five ESCC patients getting NICT were included in the study. With a mean age of 64 years (range: 45–75 years), there were 181 (88.3%) males and 24 (11.7%) females. Of the 205 cases, ypT stages T0, T1-2, and T3-4a were present in 41 (18.8%), 141 (64.7%), and 36 (16.5%) of the cases, respectively. The McKeown approach was performed on 175 (85.4%) patients, while the Ivor Lewis technique was performed on the remaining 30 (14.7%) patients. In terms of NICT regimens, camrelizumab, pembrolizumab, nivolumab, tislelizumab, or sintilimab were administered in 111 (54.1%), 27 (13.2%), 12 (5.9%), 40 (19.5%), and 15 (7.3%) of the cases, respectively. After surgery, 121 patients (59.0%) had lymph node metastases. Sixty-four (31.2%) individuals had pCR following NICT. The follow-up period ranged from 7 to 54 months, with a median of 40 months. Fifty-five (26.8%) instances resulted in death, and 79 (38.5%) patients experienced relapses.

### Comparisons between IIPS and other IIIs

To determine its prognostic utility, IIPS was compared against conventional hematological indices, including SIRI, SII, CAR, NLR, PLR, and LMR. **Figure 2B** displays the correlation diagram for all IIIs. The ROCs revealed that IIPS had the largest AUC (DFS=0.668 and OS=0.623) when compared to other IIIs, indicating a higher capacity for prediction (**Figures 2C, D**). In comparison to other IIIs, additionally, the DCAs endorsed the superior clinical use of IIPS in DFS and OS (**Figures 2E, F**). IIPS once more outperformed the other IIIs in terms of predictive value in the time-dependent AUCs (**Figures 2G, H**). Therefore, IIPS had the best predictive ability among all these indicators.

### Relationships between IIPS and clinical characteristics

**Figures 2I, J** illustrates the connection between IIPS and DFS/OS, suggesting a non-linear relationship. The ideal IIPS threshold was determined using an RCS model (**Figures 2K, L**). The RCS model identified 200 as the ideal IIPS cutoff point. Then, using an ideal threshold of 200, the patients were split into two groups (**Figure 2M**). The clinical characteristics grouped by IIPS are shown in **Table 1**. Except for the statistically significant differences in ypN stage (P=0.020) between the two groups, there were no statistically significant differences in the other clinical characteristics. There were no statistically significant differences between the two groups in terms of immunotherapy regimens (P=0.962) or surgical methods (P=0.225).

**Table 1:** Patient characteristics grouped by IIPS in ESCC receiving NICT.

Clinical characteristics	Total	Low IIPS (n=103)	High IIPS (n=102)	P-value
Age (median, IQR, years)	63 (57, 68)	64 (57, 69.5)	62 (57, 67.8)	0.388
Sex (female/male, %)	24/181	16 (15.5)/87 (84.5)	8 (7.8)/94 (92.2)	0.087
Smoking history (yes/no, %)	147/58	71 (68.9)/32 (31.1)	76 (74.5)/26 (35.5)	0.375
Drinking history (yes/no, %)	147/58	69 (67.0)/34 (33.0)	78 (76.5)/24 (23.5)	0.132
BMI (mean ± SD, Kg/m2)	21.60 ± 1.79	21.65 ± 1.81	21.55 ± 1.77	0.683
Tumor location (n, %)				0.162
upper	20	10 (9.7)	10 (9.7)	
middle	121	67 (65.0)	54 (52.9)	
lower	64	26 (25.3)	38 (37.3)	
Surgical method (M/I, %)	175/30	91 (88.3)/12 (11.7)	84 (82.4)/18 (17.6)	0.225
Differentiation (n, %)				0.942
well	47	24 (23.3)	23 (22.5)	
moderate	88	45 (43.7)	43 (42.2)	
poor	70	34 (33.0)	36 (35.3)	
Immunotherapy regimen (n, %)				0.962
camrelizumab	111	57 (55.3)	54 (52.9)	
pembrolizumab	27	13 (12.6)	14 (13.8)	
nivolumab	12	5 (4.9)	7 (6.9)	
tislelizumab	40	21 (20.4)	19 (18.6)	
sintilimab	15	7 (6.8)	8 (7.8)	
Vessel invasion (yes/no, %)	30/175	13 (12.6)/90 (87.4)	17 (16.7)/85 (83.3)	0.413
Perineural invasion (yes/no, %)	37/168	18 (17.5)/85 (82.5)	19 (18.6)/83 (81.4)	0.83
Tumor length (median, IQR, cm)	1.9 (0, 3.0)	1.6 (0, 3.0)	2.0 (0, 3.1)	0.167
pCR (yes/no, %)	64/141	35 (34.0)/68 (66.0)	29 (28.4)/73 (71.6)	0.391
ypT stage (n, %)				0.149
T0	64	35 (34.0)	29 (28.4)	
T1-2	68	38 (36.9)	30 (29.4)	
T3-4a	73	30 (29.1)	43 (42.2)	
ypN stage (positive/negative, %)	84/121	34 (33.0)/69 (67.0)	50 (49.0)/52 (51.0)	0.02
Adjuvant treatment (yes/no, %)	42/163	18 (17.5)/85 (82.5)	24 (23.5)/78 (76.5)	0.283

IIPS, immune-inflammatory prognostic score; ESCC, esophageal squamous cell carcinoma; NICT, neoadjuvant immunochemotherapy; BMI, body mass index; IQR, interquartile range; SD, standard deviation; M/I, McKeown/Ivor Lewis; pCR, pathological complete response; TNM, tumor node metastasis.

### Predictors of DFS and OS

**Figure 3:**
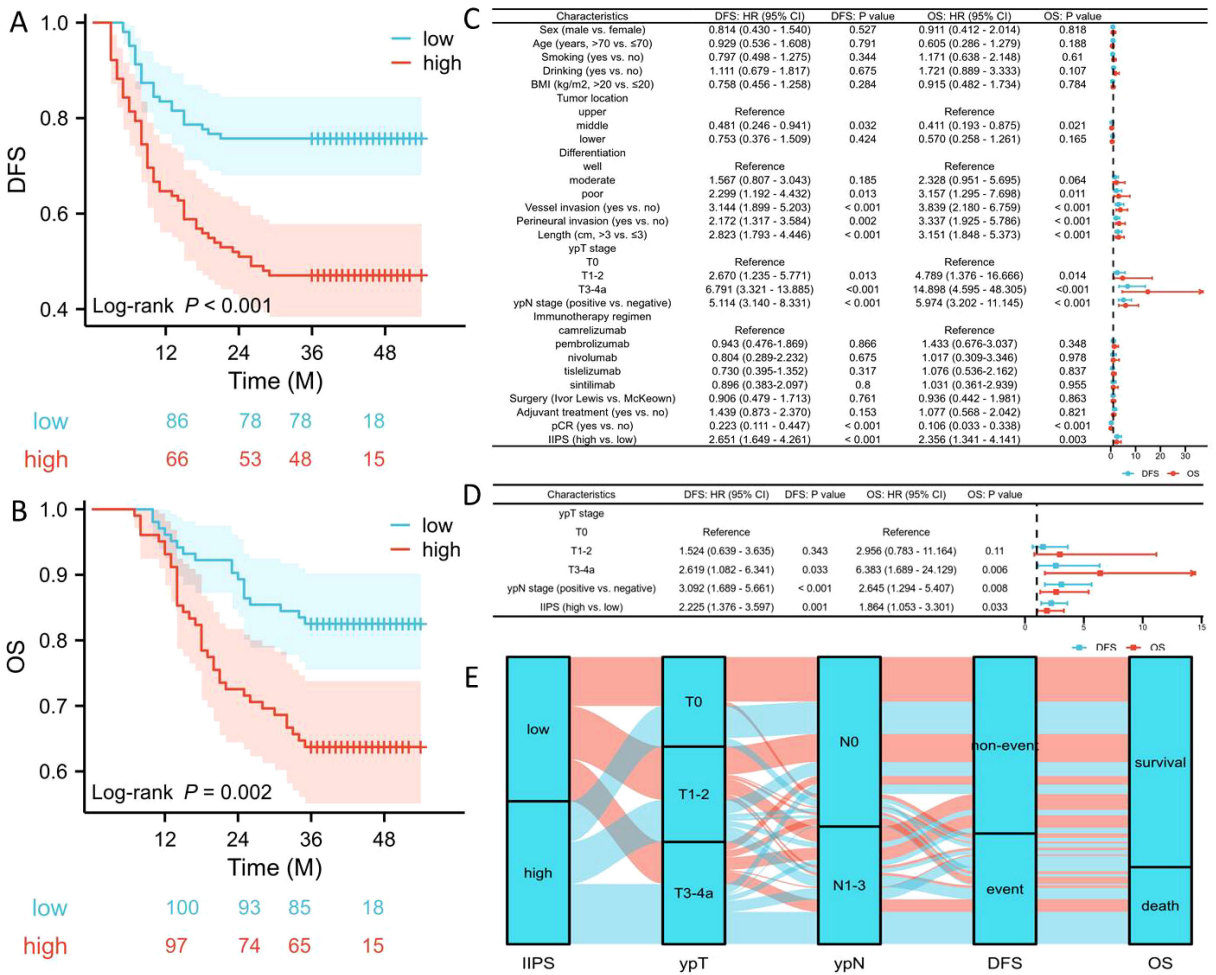
DFS **(A)** and OS **(B)** grouped by IIPS. Forest plots indicated the results of DFS and OS in univariate **(C)** and multivariate **(D)** cox regression analyses. Sankey diagram revealed the correlation regarding IIPS, ypT, ypN, and prognosis **(E)**.

Patients exhibiting high IIPS demonstrated significantly worse 3-year DFS (47.1% vs. 75.7%, P<0.001; **Figure 3A**) and OS (63.7% vs. 82.5%, P=0.002; **Figure 3B**) compared to those with low IIPS. To reduce the therapeutic heterogeneity, we analyzed the prognostic relationships between different immunotherapy regimens and surgical methods. The results revealed that there was no statistically significant difference in DFS/OS between different immunotherapy regimens (**Supplementary Figures S1A, B**) and surgical methods (**Supplementary Figures S1C, D**). Subgroup analysis revealed that IIPS provided effective prognostic stratification in different immunotherapy regimens (**Supplementary Figures S1E–H**). For the surgical methods, IIPS had the same statistical significance in the McKeown approach. Nonetheless, with the Ivor Lewis method, despite a trend of prognostic differences between the two groups, the difference was not statistically significant, possibly because of the limited sample size (only 30 patients) (**Supplementary Figures S1I–L**). **Figures 3C, D**, respectively, display the Cox regression analyses for OS and DFS. IIPS was found to be an independent predictor of both OS (HR=1.864, 95% CI=1.053-3.301, P=0.033) and DFS (HR =2.225, 95% CI=1.376-3.597, P=0.001). Compared to the low IIPS group, the high IIPS group had a 2.225-fold increased risk of recurrence and a 1.864-fold higher mortality risk. In this research, neither the surgical method (DFS: P=0.761; OS: P=0.863) nor the immunotherapy regimen (DFS: P=0.895; OS: P=0.924) was an independent prognostic factor. The association between IIPS and clinical outcomes was examined using the Sankey diagram, which showed that the group with the highest IIPS had a higher chance of mortality and recurrence in those with ESCC receiving NICT (**Figure 3E**).

## Discussion

For the first time, we showed in this study that IIPS is an independent prognostic factor from NICT in ESCC. Compared to those with low IIPS, individuals with high IIPS showed worse 3-year OS (63.7% vs. 82.5%, P=0.002) and DFS (47.1% vs. 75.7%, P<0.001). Notably, IIPS showed the highest predictive capacity for OS and DFS when compared to the most popular and well-established IIIs. Likewise, DCA curves showed better IIPS prediction values for OS and DFS. In patients with ESCC undergoing NICT, IIPS thus turned out to be the most promising potential indicator for prognostic classification.

Beyond intrinsic tumor biology, inflammatory response and immune status significantly influence cancer outcomes. Variations in treatment tolerance underscore the importance of these IIIs in therapeutic efficacy and survival (19, 20). To choose the best course of action and forecast clinical results, predictive indices must be identified prior to therapy. Inconsistent results were obtained from most investigations that assessed the immune-inflammatory status unilaterally, employing only one parameter to describe the total status. In order to represent the entire immunological and inflammatory condition, a complete index must be established. In this research, we established, for the first time, IIPS reflecting the balance of immune and inflammatory status to explore its prognostic value in patients with ESCC receiving NICT.

It is becoming well acknowledged that IIIs, which reflect the equilibrium of the host’s immunological and inflammatory condition, are crucial for the prognosis of cancer (21). IIPS serves as a comprehensive index that incorporates all of the following elements. Despite being primarily produced by hepatocytes rather than inflammatory cells, plasma CRP is the most commonly utilized biomarker of inflammation (22). CRP release is stimulated by IL-6, which also controls stromal desmoplasia, encourages tumor-induced immunosuppression and angiogenesis, suppresses apoptosis, increases the proliferation of cancer cells, and aids in metastasis, including the creation of a pro-metastatic niche in the liver (23). The primary component of serum proteins, serum ALB, has been shown to have a role in the emergence of systemic inflammation and may be utilized extensively to evaluate the nutritional status and severity of cancer patients’ diseases (24). By releasing cytokines, chemokines, and growth factors, NEUs can control the tumor microenvironment and encourage tumor migration, angiogenesis, and proliferation (25). By secreting different cytokines, MONs can be activated as tumor-associated macrophages, which encourage angiogenesis, metastasis, and invasion of tumor cells (26). PLTs interact with circulating tumor cells to produce thrombus, which help tumor cells evade immune system action. However, activated PLTs can encourage the tumor invasion and migration by the release of several physiologically active cytokines (27, 28). On the one hand, LYMs enter the tumor microenvironment and influence and eliminate tumor cell growth and metastasis. On the other hand, LYMs establish an immunological response to tumor cells and play a role in the immune control of the tumor microenvironment (29, 30).

However, the relationship between IIPS and ESCC remains unclear. For the first time, we showed in this study that IIPS is a novel and useful prognostic index from NICT in ESCC. Furthermore, IIPS is thought to be the most promising option for prognostic stratification. As a result, IIPS helped physicians better understand the overall inflammatory response and immune status, improve the prognosis of ESCC patients undergoing NICT. IIPS may therefore do a preliminary assessment of these patients’ clinical status and prognosis before beginning treatment. Although it is not mandatory, adjuvant immunotherapy is suggested at the locally advanced stage of patients with high IIPS, and close monitoring is advised in the early stages.

It is necessary to address the several limitations of this study. First of all, selection bias was unavoidable, as it is in all retrospective observational researches carried out at a single center. However, this research adopted relatively strict inclusion and exclusion criteria, which helps to reduce screening bias. Furthermore, different immunotherapy regimens and surgical methods may still result in different outcomes, which should be remembered. Therefore, we stratified according to the surgical methods and immunotherapy regimens. The results showed that there was no statistically significant difference in the surgical methods and immunotherapy regimens between the two groups of patients, thereby further indicating the homogeneity of the samples. Additionally, the results of subgroup analysis indicated that IIPS had statistical prognostic stratification significance in different surgical methods and immunotherapy regimens. Therefore, the current study has significant clinical significance for the current NICT predictive indicators and provides a direction for subsequent prospective studies at the same time. Thirdly, despite the rigorous inclusion and exclusion criteria utilized in this investigation, IIPS is derived from peripheral blood and may be impacted by a number of factors that could impact the findings. Fourthly, even though IIPS, DFS, and OS showed a strong correlation, the long-term effects have not yet been verified. Fifthly, all patients in this study lacked the detection of PD-L1. Previous studies have shown that different PD-L1 expressions have different effects on immunotherapy, thereby possibly affecting the prognosis (31, 32). Additionally, it is necessary to take into thought how the usage of antibiotics affects the prognosis of immunotherapy (33). A meta-analysis indicated that antibiotic use was significantly associated with poor survival in cancer patients treated with ICI immunotherapy, especially for those with antibiotic use in the period near the initiation of treatment (34). Furthermore, studies have also shown that nutrition-related indicators are significantly associated with the prognosis of EC patients treated with ICIs (35). Although IIPS in the current study has certain advantages when compared with some classic nutrition-related Indicators, there is still a certain degree of bias in these hematological indicators. Sixthly, it should be noted that patients’ timeliness and completeness may be impacted by treatment-related adverse events (TRAEs) during NICT, which could potentially impair the overall efficacy of therapy (36, 37). Finally, the specific mechanisms between IIPS and NICT in ESCC have not been fully clarified. Subsequently, we will further explore the relationship between IIPS and tumor microenvironment, especially the relationship with the tumor infiltrating lymphocytes and PD-L1 expression (38, 39). Thus, further and more prospective investigations are required to confirm the existing findings.

## Conclusions

In summary, the therapeutic efficacy of NICT for ESCC can be predicted by pretreatment IIPS. IIPS is an innovative, sensitive, and useful index that helps clinicians giving individualized treatments because of improved prognostic stratification.

## Data Availability

The raw data supporting the conclusions of this article will be made available by the authors, without undue reservation.

## Supplementary material

Supplementary Figure 1 DFS **(A)** and OS **(B)** grouped by immunotherapy regimens. DFS **(C)** and OS **(D)** grouped by surgical methods. Subgroup analysis regarding immunotherapy regimens in DFS **(E, F)** and OS **(G, H)**. Subgroup analysis regarding surgical methods in DFS **(I, J)** and OS **(K, L)**.
