# Supplementary figures and images for: Pretreatment immune-inflammatory prognostic score in predicting clinical outcomes in esophageal squamous cell carcinoma receiving neoadjuvant immunochemotherapy
Source: Front Immunol. 2025 Aug 6;16:1617681. doi: 10.3389/fimmu.2025.1617681 (PMC12364683; doi:10.3389/fimmu.2025.1617681)

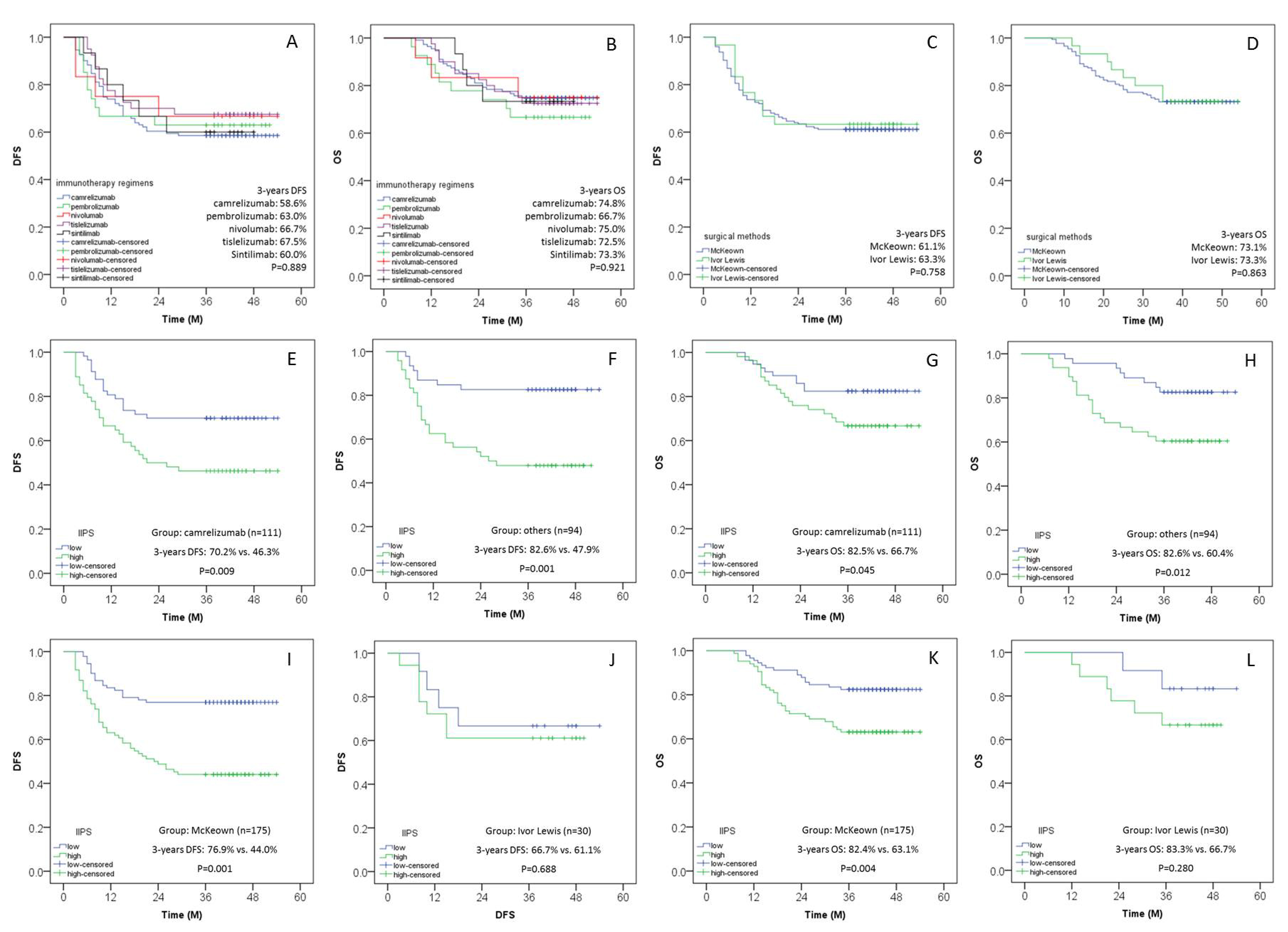

Supplement: Supplementary Figure 1 — DFS (A) and OS (B) grouped by immunotherapy regimens. DFS (C) and OS (D) grouped by surgical methods. Subgroup analysis regarding immunotherapy regimens in DFS (E, F) and OS (G, H). Subgroup analysis regarding surgical methods in DFS (I, J) and OS (K, L). [file Image1.jpeg]
